# Knowledge, attitudes, and practices (KAP) of animal health staff and farmers towards brucellosis control in sheep and goat in China: A cross-sectional study

**DOI:** 10.1371/journal.pone.0318270

**Published:** 2025-01-24

**Authors:** Quangang Xu, Alongkorn Amonsin, Saharuetai Jeamsripong

**Affiliations:** 1 Division of Epidemiology Survey, China Animal Health and Epidemiology Center, Shandong, China; 2 Faculty of Veterinary Science, Center of Excellence for Emerging and Re-emerging Infectious Diseases in Animals and One Health Research Cluster, Chulalongkorn University, Bangkok, Thailand; 3 Faculty of Veterinary Science, Department of Veterinary Public Health, Chulalongkorn University, Bangkok, Thailand; 4 Faculty of Veterinary Science, Department of Veterinary Public Health, Research Unit in Microbial Food Safety and Antimicrobial Resistance, Chulalongkorn University, Bangkok, Thailand; Federal Ministry of Agriculture and Rural Development, NIGERIA

## Abstract

**Background:**

In China, brucellosis has resurfaced recently with a discernible spatial distribution, particularly affecting dairy herds and small ruminant populations. However, limited dissemination of knowledge, attitudes, and practices (KAP) for brucellosis control exists among farmers and animal health staff. This study aimed to assess the KAP of brucellosis control and prevention in animal health staff and farmers, with the goal of educating the public regarding the application of efficient brucellosis control and prevention strategies.

**Methods:**

We conducted a cross-sectional survey involving 1,468 participants, comprising farmers (n = 1,067) and animal health staff (n = 401) based on their significant density of sheep and goat population. They completed a questionnaire targeting a specific group of farmers and animal health staff through face-to-face interviews. The KAP scores were then categorized as either satisfactory or unsatisfactory based on a cutoff set at ≥80% of the total score for satisfactory. Binary logistic regression was used to identify the association between KAP and geographical information.

**Results:**

The results indicated satisfaction with KAP among farmers (57.7%, 75.8% and 87.0%) and animal health staff (80.5%, 84.5%, and 78.3%) at ≥ 80% cut-off point of total score. The primary concern of farmers is involved in the understanding of the route of transmission and handwashing practices after contacting animals. Predictors of higher knowledge and positive attitude included province of residence and age. The factors linked to satisfactory practice levels consist of province of residence, educational level, and a history of family members’ infections (*p* < 0.05). Among animal health staff, the primary factor associated with KAP was province of residence, sex, level of education, and history of family members infection (*p* < 0.05). Additionally, brucellosis information primarily originated from traditional promotional materials and veterinarians.

**Conclusions:**

This study emphasized that the KAP scores for both animal health staff and farmers were satisfactory, except for a suboptimal knowledge score among farmers. To proactively control future brucellosis outbreaks, it is imperative to develop targeted educational resources aimed at enhancing the understanding of brucellosis, particularly among farmers. Ensuring the availability and accessibility of informative materials for the effective prevention and control of brucellosis in livestock animals.

## Introduction

Brucellosis represents a highly communicable bacterial zoonotic disease that affects a broad spectrum of humans and terrestrial animals, including cattle, buffaloes, sheep, and goats [1]. Sheep and goats serve as primary reservoirs for *Brucella melitensis* [1]. Among *Brucella* species, *B*. *melitensis* poses the greatest threat, being the most virulent and intrusive bacterial species in humans, followed by *B*. *suis* and *B*. *abortus* [2]. The clinical manifestations of brucellosis are diverse in humans and animals. Common clinical signs in humans are nonspecific, including symptoms such as fever, sweats, anorexia, headache, backache, malaise, and joint pain. Chronic cases of human brucellosis may lead to the development of conditions, such as encephalitis, meningitis, arthritis, sacroiliitis, spondylitis, osteomyelitis, and endocarditis [3]. In livestock, brucellosis has detrimental effects on reproductive performance, including abortion and infertility, resulting in reduced production levels and increased expenses related to veterinary care, resulting in a substantial socioeconomic burden on livestock production [4,5].

Regarding transmission routes, humans can be infected by *Brucella* spp. through direct contact with infected animals and their carcasses, or by consuming unpasteurized dairy products, undercooked meat, and animal derivatives sourced from infected animals [5]. Individuals with occupational exposure to livestock, such as livestock owners, abattoir workers, shepherds, and veterinarians, residing in endemic areas of brucellosis, are considered a high-risk population.

The global prevalence of brucellosis is estimated to 15.5%, with an annual report of 500,000 new human cases [6]. Incidence of human brucellosis varies based on different factors, including milk and dairy processing methods, hygiene conditions, socioeconomic status, and climate [7]. The actual number of animal brucellosis cases is approximately 10–25 times higher than the reported human cases each year [8]. In Latin America, annual losses attributable to bovine brucellosis are estimated at $600 million [9]. Between 1934 and 1997, the expenditure for the brucellosis eradication program in the United States was approximately $3.5 billion [10]. The considerable cost related to animal production, economic considerations, and public health concerns have driven the global adoption of preventive and control initiatives for brucellosis cases.

In China, the first human brucellosis was reported since 1905, with an incidence rate ranging between 0.42 and 1.0 cases per 100,000 inhabitants from 1955 to 1978. The decline in brucellosis cases was observed between 1979 and 1994. However, the disease reemerged in 1995, coinciding with an increase in the number of infected livestock [11]. Recently, brucellosis outbreak in China involving *B*. *melitensis* were identified in humans, with sheep and goats identified as the primary sources of infection [12]. Although the Chinese government implements an extensive program to prevent and control brucellosis, the disease remains a significant threat to public health and the economy.

Effective prevention and control measures directed at livestock are crucial in minimizing human brucellosis cases. It is a recommendation to consider the knowledge, attitudes, and practices (KAP) of livestock owners, workers, and veterinarians when implementing strategies for brucellosis control and prevention [13]. Therefore, the objective of this study was to assess KAP related to brucellosis prevention and control among farmers and animal health staff. Additionally, the study aimed to identify various channels through which brucellosis information is currently available and could be accessed by different occupational groups in the present and future. This study is expected to offer technical support for future interventions in human and animal health, contributing to the control and prevention of brucellosis.

## Materials and methods

### Study area

**Fig 1:**
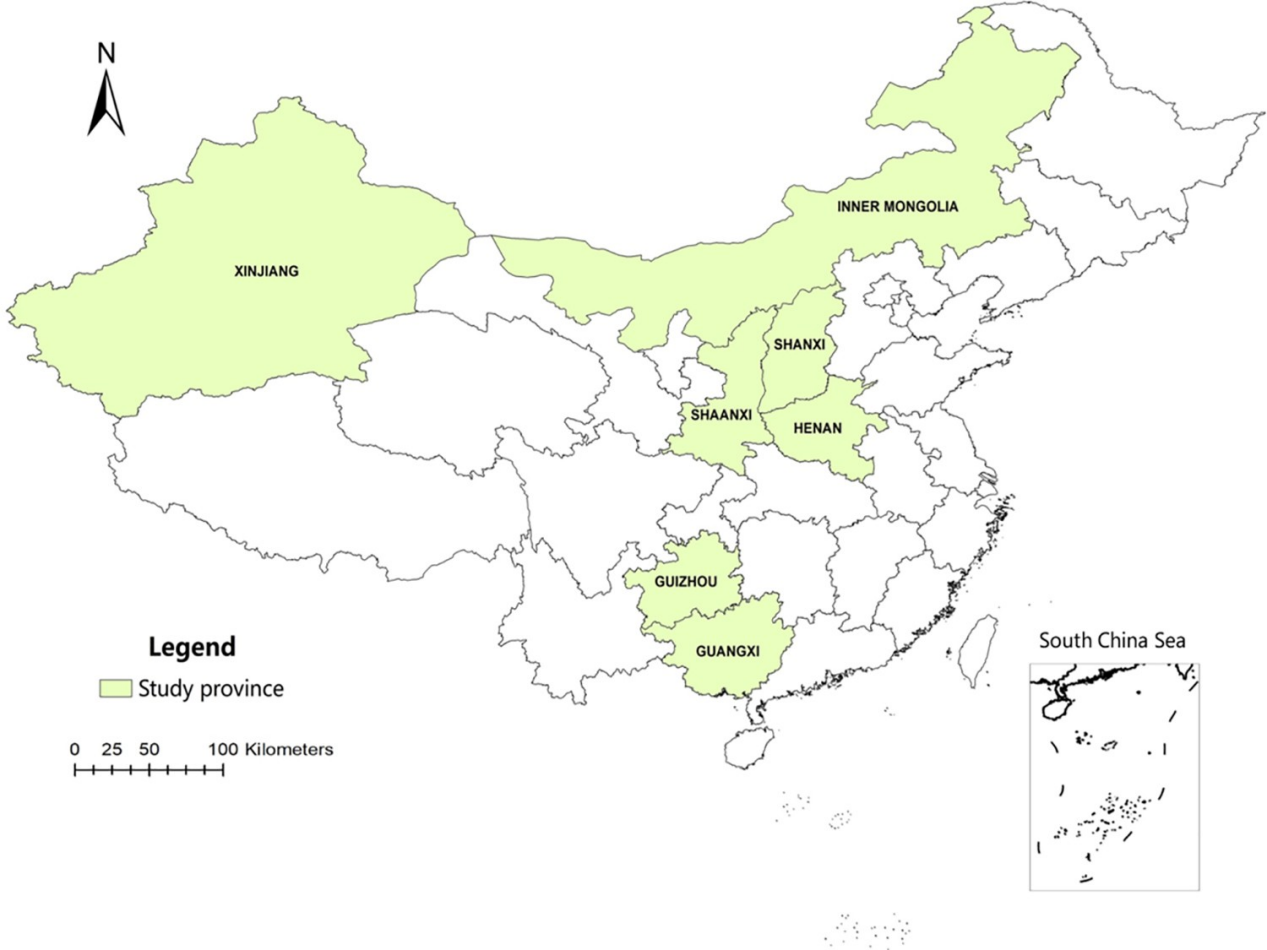
Geographic distribution of sampling sites, including Shaanxi, Shanxi, Guangxi, Xinjiang, Henan, Guizhou, and Inner Mongolia in China.

The study was conducted during April 2018 to December 2018, and covered seven provinces: Inner Mongolia, Xinjiang, Shaanxi, Shanxi, Henan, Guizhou, and Guangxi. The provinces were chosen based on their significant density of sheep and goat population (Fig 1). The total count of sheep and goats in China was approximately 297 million heads [14]. Specifically, the sheep and goats observed in this study represented 48% of the total sheep and goat populations in China. In general, ewes are bred from August to October, with lambing occurring from February to April of the following year. For fattening sheep and goats, those raised on free feeding can be slaughtered after 10–14 months, while those raised in a captive fattening system can be slaughtered after 5–8 months. Ewes used for production are generally kept for 5–6 years.

### Study design

This study was conducted as a cross-sectional survey involving farmers and community animal health staff.

### Participants

Sheep and goat farmers were defined as individuals actively involved in the practice of raising sheep or goats, possessing previous experience working with animals and expressing their willingness to share information. Community animal health staff included veterinarians and other animal health workers who demonstrated proficient Chinese language. All participants were recruited through convenience sampling. Exclusion criteria, applicable to both farmers and community animal health staff, encompassed individuals residing outside the study area and those who declined to provide information on brucellosis. The study comprised 1,067 sheep and goat farmers, along with 401 individuals from the community animal health staff.

Participants for the survey were chosen using a convenient sampling strategy, with assistance from China’s animal disease prevention and control agencies at central, provincial, prefectural, county, and township levels. The process involved selecting counties, with county agencies recommending towns. At the township level, villages/communities and farmers were identified. Households with a certain number of sheep or goats were then interviewed, with community animal health staff guiding the process. The interview team consisted of four members from the China Animal Health and Epidemiology Center, two members from the provincial Animal Disease Prevention and Control Center, and 10–15 community animal health staff in each county.

### Questionnaire

The 63-item survey for farmers and the 55-item survey for animal health staff were developed by QX and SJ with assistance from the China Animal Health and Epidemiology Center. The questionnaire’s framework has been developed with reference to these prior studies [15–17]. Demographic characteristics, including province, sex, age, educational level, and history of family infection (5 questions), were collected from fill-in questions from both farmers and animal health staff. Additionally, marital status, number of family members, religion, and farming experience (4 questions) were included only for farmers. Regarding KAP, all questionnaires consisted of yes/no answers. Knowledge included 26 questions for farmers and 25 questions for animal health staff, attitudes included 5 questions, and practices included 11 questions for farmers and 8 questions for animal health staff. Current and future media accessibility were assessed with 12 questions for both farmers and animal health staff, also based on yes/no answers. A score of “1” was assigned to every correct answer and “0” for every incorrect answer. Overall, the KAP score ranged from 0–26 for Knowledge, 0–5 for Attitudes, and 0–11 for Practices for farmers, and 0–25 for Knowledge, 0–5 for Attitudes, and 0–8 for Practices for animal health staff.

To ensure the quality of the questions, the index of item-objective congruence (IOC) was evaluated by three experts in this field, and the overall IOC value of KAP questionnaires was determined to be 0.8. Additionally, all questions were verified by three experts in the field, and pre-tests were conducted involving 20 farmers and 10 animal health staff in Shandong province. Following the pre-test phase, the questionnaires were refined based on the feedback received and any issues identified during this initial testing period. Additionally, in each county, 10–12 additional staff members from the local veterinary station joined the investigators. All investigators received China Animal Health and Epidemiology Center (CAHEC) training to ensure their ability to effectively administer the questionnaire and collect data.

### Data collection

Face-to-face interviews were conducted directly on farms with both sheep and goat farmers and community animal health staff. A total of 1,067 farmers responded to the questionnaires, resulting in a response rate of 92.4% (1,067 out of 1,155). For the community animal health staff, 401 responded to the questionnaires, indicating a participation rate of 95.5% (401 out of 420). Consequently, 1,067 farmers and 401 animal health staff were included in the data analysis, respectively.

### Data analysis

Data management, including data entry and cleaning, were conducted using Microsoft Excel before transferring the data to STATA18 (StataCorp, College Station, TX, USA). Sample sites were visually represented using ArcGIS software (Esri, CA, USA). Descriptive statistics were used to analyze demographic characteristics, KAP, and media accessibility. The KAP scores were categorized into two groups, namely satisfactory and unsatisfactory, based on a cutoff set at ≥80% for satisfactory.

Binary logistic regression analysis was conducted to examine differences in demographic characteristics for KAP scores, and variables were selected for inclusion in multivariable regression models. Both forward selection and backward elimination were utilized to identify the final model for KAP. Odds ratios (OR) and 95% confidence intervals (C.I.) were used to quantify the association between demographic variables and KAP. Likelihood ratio tests a significance level of *p* < 0.05 was applied for final regression models. Akaike Information Criterion (AIC) was used to select final models that strike a balance between model fit and the complexity of parameters. Lower AIC values indicate models that fit the data better compared to others. Pearson’s chi-squared test was conducted to assess the strength of the relationship between each pair of KAP categories. The test hypotheses were established as two-sided evaluations with *p* ≤ 0.05. All statistical analyses were performed using STATA18.

### Ethical approval

The research protocol received approval from CAHEC under the Ministry of Agriculture and Rural Affairs (CAHEC-ES-2018-001). Ethical approval for the questionnaire was obtained from the Division of Epidemiology Survey within CAHEC. Each participant provided written informed consent after being informed of the study’s objectives and procedures. In the cases where the participants faced difficulty understanding Mandarin Chinese, the staff of the local veterinary station provided clarification in the local language. All data collected in the study underwent a process of anonymization to safeguard confidentiality and privacy.

## Results

### Study population and demographic characteristics of farmers

Farmers in the study were drawn from seven provinces in China, consisting of Shanxi (27.6%), Henan (14.5%), Shaanxi (13.8%), Inner Mongolia (14.2%), Guizhou (14.1%), Xinjiang (9.4%) and Guangxi (6.6%), respectively. The majority of the farmers were male (80.1%), aged between 46 and 60 years (52.4%), and were married (91.8%) (Table 1). More than 80% of the households consisted of 1 to 5 family members. About 76.2% of the participants completed junior secondary school, with an additional 16.1% having completed senior high school. Furthermore, the majority of farmers (70.7%) indicated that they did not follow any religious affiliation. More than half of the farmers (65.1%) had been involved in goat and sheep farming for more than 10 years. Within the last five years, 12.9% of the family members of farmers had contracted *Brucella* infections.

**Table 1:** Univariable analysis between demographic distribution and knowledge, attitudes, and practices among farmers (n = 1,067).

Parameter	n (%)	Knowledge			Attitudes			Practices		
		OR	95% CI	*p*	OR	95% CI	*p*	OR	95% CI	*p*
Province Shaanxi Shanxi Guangxi Xinjiang Henan Guizhou Inner Mongolia	147 (13.8) 294 (27.6) 70 (6.6) 100 (9.4) 155 (14.5) 150 (14.1) 151 (14.2)	Ref. 1.08 0.28 2.03 0.49 0.30 0.74	0.72–1.63 0.16–0.51 1.13–3.67 0.31–0.78 0.18–0.47 0.46–1.18	0.710 <0.0001** 0.017* 0.003* <0.0001** 0.210	Ref. 2.08 0.32 1.52 0.37 0.31 0.70	1.19–3.65 0.17–0.60 0.75–3.11 0.22–0.62 0.18–0.52 0.40–1.22	0.011 <0.0001** 0.247 <0.0001** <0.0001** 0.213	Ref. 0.61 0.80 1.28 0.48 0.49 0.80	0.35–1.06 0.36–1.77 0.67–2.42 0.24–0.95 0.25–0.99 0.43–1.50	0.080 0.583 0.453 0.036* 0.047* 0.488
Sex Male Female Unspecified	854 (80.1) 74 (6.9) 139 (13.0)	Ref. 0.92 0.80	0.57–1.49 0.45–1.41	0.728 0.442	Ref. 0.75 0.54	0.41–1.38 0.27–1.07	0.357 0.077	Ref. 0.66 1.42	0.34–1.27 0.68–2.98	0.216 0.350
Age (years) 18–30 31–45 46–60 >60 Unspecified	38 (3.5) 322 (30.2) 559 (52.4) 125 (11.7) 23 (2.2)	Ref. 0.52 0.34 0.42 0.58	0.24–1.14 0.16–0.72 0.18–0.96 0.19–1.82	0.103 0.005* 0.041* 0.351	Ref. 0.56 0.42 0.46 0.55	0.21–1.48 0.16–1.10 0.16–1.28 0.14–2.14	0.239 0.077 0.137 0.085	Ref. 1.10 0.63 0.73 1.12	0.44–2.75 0.25–1.57 0.26–2.03 0.28–4.49	0.841 0.320 0.543 0.870
Marital status Married Unmarried Unspecified	979 (91.8) 31 (2.9) 57 (5.3)	Ref. 0.92 1.21	0.45–1.89 0.70–2.09	0.820 0.504	Ref. 1.10 1.09	0.47–2.59 0.58–2.05	0.825 0.797	Ref. 1.02 1.65	0.35–2.97 0.83–3.27	0.969 0.152
Number of family members (person) ≤ 5 > 5	877 (82.1) 190 (17.9)	Ref. 0.60	0.44–0.83	0.002*	Ref. 0.54	0.39–0.76	<0.0001**	Ref. 1.60	1.05–2.45	0.029*
Educational level Junior secondary school or lower secondary school Senior high school College or above Unspecified	813 (76.2) 172 (16.1) 30 (2.8) 52 (4.9)	Ref. 1.45 1.95 1.88	1.03–2.03 0.88–4.31 1.03–3.44	0.034* 0.099 0.041*	Ref. 1.42 1.35 0.92	0.94–2.14 0.54–3.35 0.49–1.72	0.095 0.519 0.784	Ref. 2.33 0.88 1.03	1.53–3.53 0.26–2.96 0.43–2.49	<0.0001** 0.839 0.939
Religion Buddhism Islam Catholicism Other No religion Unspecified	52 (4.9) 89 (8.3) 12 (1.1) 18 (1.7) 754 (70.7) 142 (13.3)	Ref. 1.35 0.79 1.04 0.16 1.29	0.67–2.70 0.23–2.79 0.59–1.82 0.04–0.61 0.68–2.46	0.403 0.718 0.903 0.008* 0.435	Ref. 0.89 1.84 1.21 1.29 1.08	0.42–1.92 0.36–9.47 0.64–2.28 0.36–4.59 0.53–2.23	0.771 0.465 0.557 0.695 0.825	1.11 0.50 0.78 0.32 0.90	0.44–2.84 0.06–4.43 0.36–1.72 0.04–2.79 0.37–2.19	0.820 0.533 0.541 0.304 0.820
Farming experience (year) ≤ 10 > 10 Unspecified	334 (31.3) 695 (65.1) 38 (5.6)	Ref. 1.13 2.34	0.87–1.46 1.10–4.97	0.377 0.027*	Ref. 1.22 1.63	0.91–1.64 0.69–3.84	0.186 0.261	Ref. 0.57 0.55	0.40–0.83 0.19–1.60	0.003* 0.273
History of family infection Yes No Unspecified	138 (12.9) 813 (76.2) 116 (10.9)	Ref. 1.27 0.89	0.88–1.83 0.54–1.46	0.195 0.645	Ref. 1.31 1.08	0.87–1.96 0.62–1.88	0.192 0.785	Ref. 0.68 0.59	0.42–1.11 0.29–1.24	0.122 0.167

Ref.: reference group; OR: odds ratio

*: *p*-value < 0.05

**: *p*-value < 0.0001.

### Knowledge, attitudes, and practices of farmers

In general, farmers received a satisfied knowledge score of 57.1% (Table 2). Most farmers (82.8%) demonstrated familiarity with the concept of brucellosis, and they believed that animals could be infected with *Brucella* (95.5%) (Table 3). Notably, a high percentage of participants (78.4%) believed in the susceptibility of sheep and goats to brucellosis. Additionally, a substantial majority (72.3%) stated the potential *Brucella* transmission from animals to humans. Respondents also indicated widespread agreement of disposal of aborted fetuses (72.5%) causing brucellosis. Regarding symptoms in infected sheep and goats, abortion (77.6%) was the most frequently observed. Farmers reported common human transmission means, such as handling aborted fetuses (77.2%), and consumption of raw meat (64.7%). Approximately, two-thirds of the participants (66.0%) expressed the belief that brucellosis could be prevented.

**Table 2:** Knowledge, attitude, and practice scores for farmers (n = 1,067) and animals health staff (n = 401).

	Overall KAP score			
KAP	Farmers		Animal health staff	
	Satisfactory (%)	Unsatisfactory (%)	Satisfactory (%)	Unsatisfactory (%)
Knowledge	609 (57.1)	458 (42.9)	319 (80.5)	82 (20.5)
Attitudes	809 (75.8)	258 (24.2)	339 (84.5)	62 (15.5)
Practices	928 (87.0)	139 (13.0)	314 (78.3)	87 (21.7)

Satisfactory and unsatisfactory were classified based on a cutoff set at ≥80% for satisfactory.

**Table 3:** Knowledge of brucellosis among farmers (n = 1,067) animal heath staff (n = 401).

Knowledge	Farmers (%)	Animal health staff (%)
You have heard of brucellosis. Yes No	883 (82.8) 184 (17.2)	352 (87.8) 49 (12.2)
Animals could be infected with *Brucella*. Yes No Unspecified	1,019 (95.5) 38 (3.6) 10 (0.9)	NA
Animals could be infected with Brucella* Cattle Sheep and goats Pig Dog	688 (64.5) 837 (78.4) 287 (26.9) 127 (11.9)	336 (83.8) 345 (86.0) 226 (56.4) 120 (29.9)
*Brucella* can spread from animal to human. Yes No Unspecified/do not know	772 (72.3) 20 (1.9) 275 (25.8)	NA
Route of sheep and goat infected with Brucella* Feeding from infected sheep and goats Unquarantined animals Randomly discard aborted fetuses Not disinfected with lambing areas	743 (69.6) 699 (65.5) 773 (72.5) 726 (68.0)	339 (84.5) 343 (85.5) 340 (84.8) 334 (83.3)
Symptoms of sheep and goat infected with Brucella* Abortion of female sheep and goats^a^ Orchitis of male animals Placenta retention Joint swelling	828 (77.6) 709 (66.5) 673 (63.1) 662 (62.0)	350 (87.3) 328 (81.8) 307 (76.6) 313 (78.1)
Route of human infected with Brucella* Contact with aborted fetuses Contact with fur Ingestion of raw milk Consumption of raw meat Respiratory transmission	824 (77.2) 547 (51.3) 601 (56.3) 690 (64.7) 528 (49.5)	347 (86.5) 306 (76.3) 321 (80.1) 339 (84.5) 283 (70.6)
Symptoms of human brucellosis* Undulant fever Sweating Asthenia Joint pain Myalgia	744 (69.7) 692 (64.9) 771 (72.3) 743 (69.6) 658 (61.7)	338 (84.3) 330 (82.3) 347 (86.5) 339 (84.5) 302 (75.3
Brucellosis can be prevented. Yes No Unspecified/do not know	704 (66.0) 15 (1.4) 348 (32.6)	334 (83.3) 67 (16.7) 0

Note

*Multiple answers allowed. NA: not available

^a^Period of abortion occurs during the last trimester of pregnancy.

Sheep and goat farmers received a grand total favorable attitude score of 75.8% (Table 4). The majority of the respondents showed positive attitudes toward the control and prevention of brucellosis. A significant portion of the participants (77.7%) expressed a desire to obtain information on strategies for control and prevention. Approximately 71.5% of the respondents showed awareness that brucellosis poses a significant threat to animal health. The comprehensive practice score was satisfied at 87.0% (Table 4). In terms of personal protective equipment (PPE), the most widely adopted measure was the use of gloves (63.2%), followed by protective eyewear (36.7%) and masks (34.5%).

**Table 4:** Proportion of attitudes and practices of brucellosis among farmers (n = 1,067) and animal heath staff (n = 401).

Factor	Proportion (%)	
**Attitudes** *	**Farmers**	**Animal health staff**
Brucellosis seriously harms the health of sheep and goats	763 (71.5)	326 (81.3)
Need to prevent human brucellosis	804 (75.4)	327 (81.5)
Need to prevent sheep and goat brucellosis	825 (77.3)	341 (85.0)
Sheep and goats need to be vaccination	822 (77.0)	342 (85.3)
Accept information on brucellosis prevention and control	829 (77.7)	344 (85.8)
**Practices** *	**Farmers**	**Animal health staff**
PPE used at work* Mask Rubber gloves Rubber shoes Protective clothing Protective glasses	368 (34.5) 674 (63.2) 283 (26.5) 212 (19.9) 392 (36.7)	343 (85.5) 341 (85.0) 340 (84.8) 336 (83.8) 300 (74.8)
Wash hands after working and contacting with animals	430 (40.3)	347 (86.5)
Management of sheep and goat carcasses Use safe disposal Sold carcasses Leave the carcass in farm Disregard/throw away Do not know Unspecified	690 (64.7) 39 (3.7) 29 (2.7) 113 (10.6) 184 (17.2) 12 (1.1)	NA
Management of aborted fetal membranes Use safety disposal Sold carcasses Leave the carcass in farm Disregard/throw away Do not know Unspecified	671 (62.9) 3 (0.3) 39 (3.7) 157 (14.7) 184 (17.2) 13 (1.2)	NA
Quarantined before flock mixed	454 (42.5)	NA
Separate raw and cooked cutting board	374 (35.1)	243 (60.6)
Separate raw and cooked knives	367 (34.4)	241 (60.1)

*Multiple answers allowed. NA: not available.

Regarding animal carcass management on farms, 64.7% of farmers indicated practicing safe disposal. Typically, safe methods for disposing of animal carcasses include burial, incineration, composting, and other approved techniques. However, 10.6% and 17.2% of farmers did not adhere to proper practices for handling animal carcasses and lacked knowledge on appropriate management, respectively. Examples of recommended procedures for managing animal carcasses involve employing PPE, ensuring proper disposal, conducting thorough cleaning and disinfection, minimizing cross-contamination, ensuring safe transportation, and providing training and awareness. Regarding the handling of aborted fetal membranes, the majority reported using safe disposal methods. However, a minority of respondents, 14.7% and 17.2% respectively, neglected proper management of aborted fetal membranes or lacked knowledge about how to handle it appropriately. Approximately 42.5% of farmers implemented quarantine measures for new animals before integrating them into the flocks. Additionally, approximately one-third of the farmers adopted the practice of using separate knives and cutting boards for raw and cooked meals within their households.

### Multiple channels for accessing information of brucellosis among farmers

**Fig 2:**
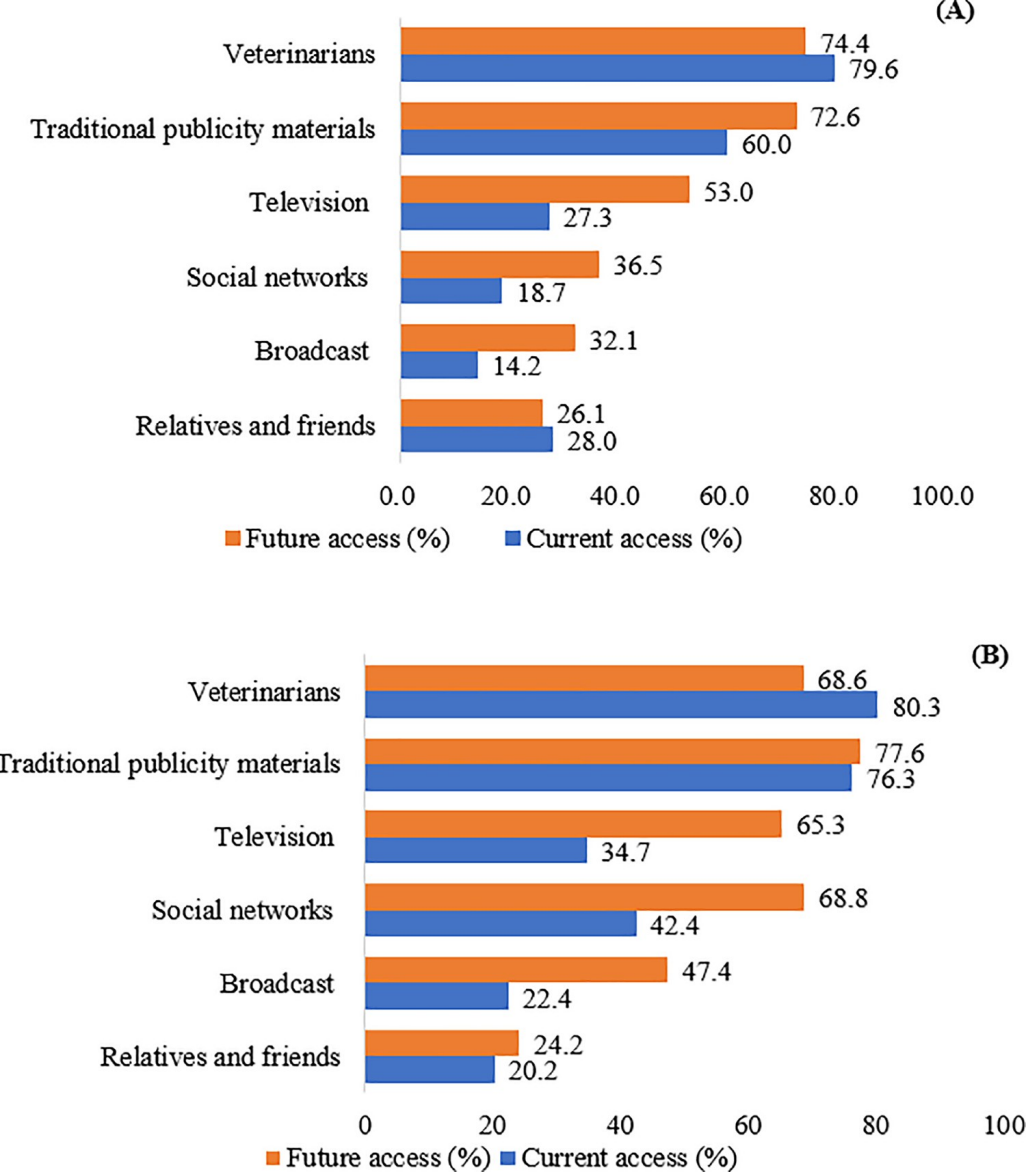
Distribution of access to brucellosis information among a) sheep and goat farmers (n = 1,067) and b) community animal health staff (n = 401).

The current and future of accessing information regarding brucellosis was classified in (Fig 2A). Farmers primarily acquired their knowledge of prevention and control from veterinarians (79.6%) and traditional informational materials (60.0%). Traditional informational materials include printed materials such as paper, books, and reports, which are not accessed electronically. For future information access, veterinarians (74.4%) and traditional informational materials (72.6%) remain the main sources for acquiring brucellosis-related knowledge. Notably, there has been a notable increase in the proportion of people seeking brucellosis information through various channels. The trend of television, the internet, and radio has seen a substantial increase from 27.3%, 18.7%, and 14.2% to 53.0%, 36.5%, and 32.1%, respectively.

### Study population and demographic characteristics of animal health staff

Survey participants came from various regions, including Shanxi (25.4%), Henan (18.7%), Guangxi (12.0%), Shaanxi (12.7%), Inner Mongolia (11.5%), Guizhou (11.2%) and Xinjiang (8.5%) (Table 5). Most of the animal health staff were male (68.6%) and in the age group 46–60 years (82.3%). Approximately 67.3% held a college degree or a higher level of education. A substantial majority (85.8%) reported that their family members had not experienced brucellosis, with 3.5% indicating their family members being infected within the previous five years.

**Table 5:** Univariable analysis of the association between demographic distribution and knowledge, attitudes, and practices among animal health staff (n = 401).

Parameter	n (%)	Knowledge				Attitudes				Practices		
		OR	95% CI	*p*	OR		95% CI	*p*	OR		95% CI	*p*
Province Shaanxi Shanxi Guangxi Xinjiang Henan Guizhou Inner Mongolia	51 (12.7) 102 (25.4) 48 (12.0) 34 (8.5) 75 (18.7) 45 (11.2) 46 (11.5)	Ref. 0.88 3.65 0.31 1.16 1.58 0.64	0.37–2.11 0.92–14.46 0.11–0.83 0.45–2.98 0.51–4.88 0.25–1.67	0.782 0.065 0.020* 0.757 0.425 0.364	Ref. 0.57 1.83 0.17 0.89 1.71 0.50		0.20–1.64 0.41–8.14 0.06–0.55 0.28–2.85 0.38–7.61 0.17–1.59	0.297 0.428 0.003* 0.850 0.483 0.241	Ref. 0.47 1.34 0.25 0.29 0.34 0.40		0.17–1.34 0.34–5.34 0.08–0.82 0.10–0.84 0.11–1.05 0.13–1.23	0.157 0.677 0.023* 0.023* 0.060 0.109
Sex Male Female Unspecified	275 (68.6) 88 (21.9) 38 (9.5)	Ref. 1.62 0.64	0.91–2.88 0.28–1.46	0.102 0.291	Ref. 0.96 0.39		0.48–1.92 0.15–0.98	0.898 0.045*	Ref. 0.69 0.32		0.37–1.31 0.14–0.78	0.260 0.011*
Age (years) 18–30 31–45 46–60 > 60 Unspecified	41 (10.2) 0 (0) 330 (82.3) 6 (1.5) 24 (6.0)	Ref. - 1.48 1.61 0.32	0.69–3.19 0.17–15.49 0.11–0.94	0.315 0.679 0.039*	Ref. - 1.34 1.0 0.24		0.56–3.20 - 0.08–0.76	0.513 - 0.015*	Ref. - 0.78 1.0 0.24		0.33–1.83 - 0.08–0.76	0.567 - 0.015*
Educational level Junior secondary school or lower secondary school Senior high school College or above Unspecified	18 (4.5) 78 (19.5) 270 (67.3) 35 (8.7)	Ref. 3.10 4.60 0.76	1.05–9.13 1.71–12.36 0.24–2.37	0.040* 0.002* 0.630	Ref. 3.89 6.24 0.54		1.24–12.15 2.22–17.52 0.17–1.71	0.020* 0.001* 0.291	Ref. 3.88 5.28 0.84		1.32–11.35 1.98–14.06 0.27–2.63	0.014* 0.001* 0.767
History of family infection Yes No Unspecified	14 (3.5) 344 (85.8) 43 (10.7)	Ref. 0.98 0.07	0.21–4.51 0.01–0.37	0.979 0.002*	Ref. 2.96 0.11		0.78–11.22 0.03–0.50	0.110 0.003*	Ref. 0.40 0.03		0.05–3.09 0.004–0.28	0.376 0.002*

Ref.: reference group; OR: odds ratio

*: *p*-value < 0.05.

### Knowledge, attitudes, and practices of animal health staff

The overall score of satisfied levels of knowledge was 57.1% (Table 2). Most animal health staff (80.5%) are familiar with brucellosis (Table 3). Survey participants believed that various animals could be infected with *Brucella*, indicating a percentage of susceptibility in sheep and goats (86.0%) and cattle (83.8%). More than 80.0% of animal health staff were familiar with the route of transmission. Most participants identified various sources of infections such as feeding infected sheep and goats (85.5%), animals not subjected to quarantine (85.5%), and disposal of aborted fetuses without proper precautions (84.8%). Considering the clinical signs of the disease of sheep and goats, the proportion including abortion (87.3%) was highest reported. The animal health staff highlighted that the primary route of human brucellosis was through contact with aborted fetuses (86.5%). Two-thirds (83.3%) of them believed that brucellosis can be prevented.

The prevalence of satisfied attitudes among animal health staff was 84.5% (Table 2). Most of them (85.3%) expressed that brucellosis represents a significant health concern for the sheep and goat populations (Table 4). Regarding the prevention and control of brucellosis, they believe that it is essential to address both human (81.6%) and animal brucellosis (85.0%). They also agreed that it is recommended for sheep and goats to receive vaccination against *Brucella* spp. in their livestock (85.3%). Most respondents (85.8%) expressed their interest in accessing information on the control and prevention of brucellosis. The satisfied score of practices observed among animal health staff was 78.3% (Table 2). The use of PPE and sanitation practices among community animal health staff is presented (Table 4). More than 85% of them practiced handwashing after contacting animals.

### Multiple channels for accessing information of brucellosis among animal health staff

Currently, the main sources of information concerning brucellosis prevention and control were veterinarians (80.3%) and traditional promotional materials (76.3%) (Fig 2B). The anticipated future trend for accessing brucellosis information continued to include traditional promotional materials (77.6%) and veterinarians (68.6%). However, there was a notable increase in the proportion of gaining knowledge of brucellosis through social networks, television, and radio broadcast, with the number rising from 42.4%, 34.7% and 22.4% to 68.8%, 65.3%, and 47.4%, respectively.

### Factors affecting knowledge, attitudes, and practices among farmers and animal health staff

Binary logistic regression analysis revealed a significant association between knowledge and several factors of farmers, including province, age, and religion (Table 6). Attitudes were associated with provinces, sex, age, and religion, while practices were associated with province, educational level and farming experience (*p* < 0.05) (Table 6). Among animal health staff, province, educational level, and history of family infection in the past five years were statistically associated with KAP (*p* < 0.05) (Table 7). Additionally, the relationship between KAP was analyzed, resulting in a statistical significance with *p* < 0.0001 (Table 8).

**Table 6:** Multivariable analysis between demographic distribution and knowledge, attitudes, and practices of farmers (n = 1,067).

Parameter	Odds ratio	95% Confidence Interval	*p*-value
**Knowledge** Province Shaanxi Shanxi Guangxi Xinjiang Henan Guizhou Inner Mongolia Age (years) 18–30 31–45 46–60 > 60 Unspecified Religion Buddhism Islam Catholicism Other religion No religion Unspecified Constant AIC = 1,361.11	Ref. 1.14 0.24 3.68 0.54 0.26 0.70 Ref. 0.46 0.24 0.27 0.45 Ref. 0.33 0.65 0.89 0.17 1.01 6.88	0.74–1.76 0.13–0.44 1.60–8.46 0.33–0.87 0.16–0.43 0.42–1.16 0.20–1.07 0.11–0.56 0.11–0.66 0.13–1.58 0.13–0.85 0.17–2.52 0.47–1.68 0.04–0.68 0.49–2.07 2.36–20.09	0.542 < 0.0001** 0.002* 0.012* < 0.0001** 0.165 0.073 0.001* 0.004* 0.213 0.022* 0.543 0.713 0.013* 0.978 < 0.0001**
**Attitudes** Province Shaanxi Shanxi Guangxi Xinjiang Henan Guizhou Inner Mongolia Sex Male Female Unspecified Age (years) 18–30 31–45 46–60 > 60 Unspecified Religion Buddhism Islam Catholicism Other religion No religion Unspecified Constant AIC = 1,076.55	Ref. 2.45 0.30 4.52 0.42 0.25 0.73 Ref. 0.71 0.42 Ref. 0.49 0.30 0.27 0.36 Ref. 0.21 1.38 0.91 1.42 0.62 21.24	1.37–4.40 0.16–0.57 1.71–11.96 0.24–0.73 0.15–0.44 0.40–1.33 0.37–1.34 0.20–0.87 0.18–1.37 0.11–0.83 0.09–0.79 0.08–1.56 0.07–0.59 0.24–7.85 0.45–1.82 0.37–5.49 0.28–1.37 5.21–86.54	0.003* < 0.0001** 0.002* 0.002* < 0.0001** 0.303 0.290 0.019* 0.176 0.020* 0.017* 0.171 0.003* 0.718 0.780 0.610 0.237 < 0.0001**
**Practices** Province Shaanxi Shanxi Guangxi Xinjiang Henan Guizhou Inner Mongolia Educational level Junior secondary school or lower secondary school Senior high school College or above Unspecified Farming experience (year) ≤ 10 > 10 Unspecified Constant AIC = 808.14	Ref. 0.56 0.55 1.51 0.33 0.41 0.60 Ref. 2.10 0.65 0.94 Ref. 0.48 0.26 0.34	0.33–1.02 0.24–1.26 0.76–2.99 0.16–0.69 0.20–0.84 0.31–1.16 1.36–3.23 0.19–2.28 0.38–2.32 0.32–0.72 0.08–0.85 0.19–0.59	0.060 0.158 0.238 0.003* 0.014 0.130 0.001* 0.505 0.891 < 0.0001** 0.026* < 0.0001**

Ref: reference group

*: *p*-value < 0.05

**: *p*-value < 0.0001: AIC: Akaike information criterion.

**Table 7:** Multivariable analysis between demographic distribution and knowledge, attitudes, and practices among animal health staff (n = 401).

Parameter	Knowledge			Attitudes			Practices		
	OR	95% CI	*p*	OR	95% CI	*p*	OR	95% CI	*p*
Province Shaanxi Shanxi Guangxi Xinjiang Henan Guizhou Inner Mongolia	Ref. 1.25 7.04 0.34 4.14 1.87 0.69	0.47–3.38 1.40–35.30 0.11–1.05 1.21–14.19 0.53–6.54 0.24–2.01	0.654 0.018* 0.060 0.024* 0.328 0.499	Ref. 0.87 4.57 0.15 4.76 0.45 0.29	0.23–3.39 0.61–34.2 0.04–0.63 0.92–24.7 0.38–14.2 0.10–1.97	0.846 0.139 0.010* 0.064 0.366 0.291	Ref. 0.55 1.87 0.26 0.48 0.30 0.39	0.17–1.74 0.40–8.75 0.07–0.97 0.14–1.66 0.09–1.03 0.11–1.40	0.306 0.426 0.045* 0.246 0.056 0.150
Educational level Junior secondary school or lower secondary school Senior high school College or above Unspecified	Ref. 9.60 13.34 4.00	2.61–35.23 3.76–47.26 0.89–17.98	0.001* <0.0001** 0.070	Ref. 24.1 27.9 3.8	4.90–118.7 6.29–123.5 0.71–20.4	<0.0001** <0.0001** 0.120	Ref. 6.11 6.12 1.67	1.86–20.13 1.92–19.56 0.42–6.70	0.003* 0.002* 0.466
History of family infection Yes No Unspecified	Ref 0.36 0.02	0.06–2.27 0.003–0.17	0.279 <0.0001**	Ref. 1.25 0.03	0.22–7.03 0.004–0.22	0.801 0.001*	Ref. 0.26 0.03	0.03–2.45 0.002–0.26	0.240 0.002*
Constant	1.24	0.15–10.14	0.084	0.55	0.05–5.62	0.610	7.93	0.67–94.4	0.101

AIC for knowledge = 330.0; AIC for attitudes = 234.94, AIC for practices = 358.61.

Ref.: reference group; OR: odds ratio

*: *p*-value < 0.05

**: *p*-value < 0.0001, AIC: Akaike information criterion.

**Table 8:** The association between knowledge, attitudes, and practices among farmers and animal health staff.

Respondents		Knowledge	Attitude	Practice
Farmers (n = 1,067)	Knowledge	-	< 0.0001	< 0.0001
	Attitudes	< 0.0001	-	< 0.0001
	Practices	< 0.0001	< 0.0001	-
Community animal health staff (n = 401)	Knowledge	-	< 0.0001	< 0.0001
	Attitudes	< 0.0001	-	< 0.0001
	Practices	< 0.0001	< 0.0001	-

## Discussion

This is the first large-scale epidemiological study conducted in China to investigate knowledge and awareness of occupational risk groups, highlighting the importance of implementing effective prevention and control measures for brucellosis. The results of this study demonstrated that most farmers and animal health staff were aware of brucellosis, which is consistent with previous studies conducted in many countries [13,18,19]. Despite the high awareness of brucellosis, understanding transmission, clinical symptoms, and prevention of brucellosis were limited due to their understanding in these specific areas is lacking. This may be because people may know that brucellosis exists but lack detailed knowledge about how it is transmitted, its clinical symptoms, and preventive measures. This can happen if general awareness campaigns focus on raising the profile of the disease without addressing the specific aspects of its transmission, symptoms, and prevention. Additionally, there may be gaps in education or training for both the general public and healthcare providers regarding the specific details of brucellosis. This could result from insufficient educational resources, ineffective communication strategies, or a lack of emphasis on these critical aspects in public health messages.

Although more than 70% of the farmers were aware that brucellosis is a zoonosis, a higher percentage (~65.5%) correctly identified the mode of transmission in animals compared to humans (~49.5%). Since sheep and goats are crucial assets for their families, serving as the primary source of income and a key means to enhance living conditions, they prioritize the health of their livestock over their own health. Various studies have emphasized that having a solid understanding of disease transmission does not necessarily guarantee a higher likelihood of adopting appropriate measures against *Brucella* infections. For instance, a study conducted in the Kyrgyz Republic indicated that farmers with a good knowledge of disease transmission routes had a potential impact on the prevention and control of brucellosis [20]. Similarly, a case-control study in Iran revealed that being informed about the route of transmission, such as the consumption of unpasteurized cheese made from raw milk, was associated with a reduced risk of contracting human brucellosis [21]. This finding suggests that improving farmers’ understanding of the disease and modes of transmission could potentially decrease the likelihood of a zoonotic transmission of brucellosis.

All participants in this study exhibited high awareness scores of brucellosis, which agreed with previous studies among health workers in Sri Lanka and Uganda [22,23]. However, previous studies indicated limited awareness among farmers in Kenya and Thailand [24,25]. These livestock producers also play a crucial role in the successful implementation of disease intervention measures [26]. In this study, most farmers and animal health staff exhibited favorable attitudes toward the prevention and control of brucellosis. Specifically, they believed that prevention and control measures should be implemented for brucellosis and demonstrated a willingness to vaccinate their livestock and expressed interest in gaining more knowledge about brucellosis.

Inappropriate practices, such as failure to isolate infected animals, insufficient vaccination, inadequate hygiene practices, lack of stringent biosecurity measures, improper disposal of animal carcasses, related to the control and prevention of brucellosis presented a notable threat of human infection [23]. This study found that the protective measures taken by farmers against brucellosis, ranging from 19.9% to 63.2%, were insufficient because they did not use protective cloth, rubber shoes, and failed to wash their hands after touching animals. Although PPE among animal health staff was relatively high, ranging from 74.8% to 85.5%, the inadequate adherence to these practices raises concerns that may pose a potential risk of human contracting zoonotic diseases. Given that brucellosis can be transmitted directly from aborted fetuses and their secretions to humans, failure to address these practices increases the risk of humans to acquire the infection [27]. Approximately 60% of farmers in this study exhibited appropriate practices in the disposal of sheep and goat carcasses and handling aborted fetal membranes, which was similar to a previous study [13]. Similarly, a previous study indicated that participants with higher levels of education demonstrated commendable practices in the prevention and control of brucellosis [28]. However, only 42.5% of farmers took the step of quarantining newly acquired sheep and goats before introducing them to the herd, a behavior previously identified as a main risk factor for transmission of brucellosis in sheep and goats [29]. Farm biosecurity can effectively reduce the potential spread of the disease to unaffected herds [30]. Another improper practice was the failure to use separate knives and cutting boards for raw and cooked meat at home. This highlights the ongoing need for more effective enhancement and reinforcement of awareness regarding proper practices in preventing and controlling brucellosis.

The primary sources of information on brucellosis indicated by farmers and animal health staff in this study were veterinarians and conventional promotional materials. Additional sources identified were television, broadcast, social networks, and friends and relatives. These findings align with previous studies in South Africa, Kenya, Tajikistan and Pakistan [13,20,31,32]. This implies the need to modernize knowledge dissemination and health interventions by efficiently utilizing various online platforms for the benefit of future users. The continuous development of new media platforms is recommended to disseminate knowledge about brucellosis and promote health interventions.

In this study, several factors, including province and age, were statistically associated with knowledge among farmers. Based on multivariable analysis of the association between demographic distribution and KAP, farmers residing in Xinjiang (OR = 3.68, CI = 1.60–8.46) had significantly higher odds of knowledge compared to those from Shaanxi (Table 6). The respondents in the northern regions such as Xinjiang exhibited greater knowledge compared to those in the southern areas. This discrepancy can be attributed to the consistently reported cases of brucellosis in northern China [32]. Therefore, the government should make significant efforts to manage and control brucellosis in these regions. Additionally, farmers in Xinjiang (OR = 4.52, CI = 1.71–11.96) and Shanxi (OR = 2.45, CI = 1.37–4.40) had a favorable attitude score than those from Shaanxi (Table 6). In terms of practices, farmers in Xinjiang (OR = 0.33, CI = 0.16–0.69) had lower odds than those from Shaanxi. Therefore, areas outside Xinjiang and Shanxi should be closely monitored for KAP among farmers. For age groups, farmers aged 18–30 had higher KAP scores than other age groups, possibly due to greater concern about brucellosis. Interestingly, farmers with a senior high school education (OR = 2.10, CI = 1.36–3.23) had higher odds of proper practices than those with a junior secondary school education. Additionally, farmers with less than 10 years of livestock operations experience had higher odds (OR = 1/0.48 = 2.08, CI = 0.32–0.72) of proper practice than those with more than 10 years of experience, indicating that the new generation of farmers is more aware of the control and prevention of brucellosis.

Based on multivariable logistic regression analysis for animal health staff, province, educational level, and history of family infection were the main predictors for KAP (Table 7). Interestingly, animal health staff from Guangxi (OR = 7.04, CI = 1.40–35.3) and Henan (OR = 4.14, CI = 1.21–14.19) had significantly higher odds of a knowledge score than those from Shaanxi, which was inconsistent with the findings from farmers. Animal health staff from Shaanxi had higher odds of satisfied attitudes (OR = 1/0.15 = 6.67, CI = 0.04–0.63) and practices (OR = 1/0.26 = 3.85, CI = 0.08–0.85) scores compared to those from Xinjiang. Specifically, among animal health staff from the northern regions such as Shanxi and Xinjiang, those with a college or higher degree, and had experience in farms for less than 5 years, demonstrated a higher degree of KAP scores (Table 7). Educational level was the primary factor associated with KAP (*p* < 0.0001). Clearly, animal health staff with higher education, such as college and above, had higher odds of knowledge (OR = 13.3 CI = 3.76–47.26), attitudes (OR = 27.9, CI = 6.29–123.5), and practices (OR = 6.12, CI = 1.92–19.56) compared to those with a junior secondary school education indicating that education is essential for KAP to control and prevent brucellosis. This finding is consistent with a previous study that identified the level of education as a major factor contributing to an individual’s inclination and ability to acquire additional knowledge [33]. Other similar studies have indicated that farmers with limited education are less likely to be aware of brucellosis and are more susceptible to contracting the disease [25,34]. The subsequent phase of health intervention should focus on enhancing educational materials and minimizing the impacts of brucellosis on both livestock and households. Furthermore, the occurrence of brucellosis within a family was associated with a KAP score of controlling and preventing brucellosis. Animal health staff who had family members infected with brucellosis tended to have higher knowledge (OR = 1/0.02 = 50.0, CI = 0.003–0.17), attitude (OR = 1/0.03 = 33.3, CI = 0.004–0.22), and practice (OR = 1/0.03 = 33.3, CI = 0.002–0.26) scores than those with an unspecify stats of family infection history (Table 7). This may be because sheep infected with brucellosis resulted in financial losses and posed a substantial risk to the health of families, leaving a lasting impact on their well-being. Undoubtedly, such a situation would motivate affected families to actively seek information regarding the prevention and control of brucellosis. The relationship found between the KAP of farmers and animal health staff was noted, suggesting that attitudes and practices are influenced by knowledge and vice versa (Table 8). To effectively control brucellosis in animals and humans, it is crucial to address several aspects including transmission routes, symptoms, preventive measures, treatment options, public health risks, and regulatory requirements [35,36]. Enhancing health literacy among farmers and animal health staff can improve their capacity to prevent, detect, and manage brucellosis, thereby safeguarding the health of both humans and animals.

The limitations of this study included generalizability and data quality, which introduced bias estimation. The study aimed to assess brucellosis awareness on specific provinces. While this limited scope raises concerns about the generalizability of the results nationwide, the selected provinces represent major sheep and goat producing areas. In terms of data quality and acknowledging the potential limitations of categorizing variables, the study utilized logistic regression analysis to determine the factors influencing KAP, which has been widely used in previous research on KAP studies [37–39]. However, utilizing mixed linear regression is a viable option for analyzing quantitative data. Despite some limitations in the study, the findings still offer valuable insights for directing future brucellosis awareness campaigns and interventions.

## Conclusions

In general, the sheep and goat farmers and community animal health workers demonstrated a satisfactory level of understanding, attitudes, and practices related to brucellosis. However, there remains considerable potential to improve knowledge and adopt better practices in brucellosis control and prevention of brucellosis. This suggests that the government should continue to promote awareness and education about brucellosis targeted in regions with endemic prevalence.

## References

[1] Abd El-Wahab E.W., Hegazy Y.M., El-Tras W.F., Mikheal A., Kabapy A.F., Abdelfatah M., et al. (2019). A multifaceted risk model of brucellosis at the human-animal interface in Egypt. *Transboundary and Emerging Diseases*, 66(6), 2383–2401. doi: 10.1111/tbed.13295 31309735

[2] Głowacka P., Żakowska D., Naylor K., Niemcewicz M., Bielawska-Drózd A. (2018). *Brucella*—virulence factors, pathogenesis and treatment. *Polish Journal of Microbiology*. 67(2), 151–161. 10.21307/pjm-2018-029 30015453 PMC7256693

[3] Esmaeilnejad-Ganji S.M., Esmaeilnejad-Ganji S.M.R. (2019). Osteoarticular manifestations of human brucellosis: A review. *World Journal of Orthopedics*. 10(2), 54–62. doi: 10.5312/wjo.v10.i2.54 30788222 PMC6379739

[4] Lokamar P.N., Kutwah M.A., Atieli H., Gumo S., Ouma C. (2020). Socio-economic impacts of brucellosis on livestock production and reproduction performance in Koibatek and Marigat regions, Baringo County, Kenya. *BMC Veterinary Research*, 16(1), 61. doi: 10.1186/s12917-020-02283-w 32070337 PMC7027201

[5] Garcell H.G., Garcia E.G., Pueyo P.V., Martín I.R., Arias A.V., Alfonso Serrano R.N. (2016). Outbreaks of brucellosis related to the consumption of unpasteurized camel milk. *Journal of Infection and Public Health*, 9(4), 523–527. doi: 10.1016/j.jiph.2015.12.006 26796768

[6] Khoshnood S., Pakzad R., Koupaei M., Shirani M., Araghi A., Irani G.M., et al. (2022). Prevalence, diagnosis, and manifestations of brucellosis: A systematic review and meta-analysis. *Frontiers in Veterinary Science*, 9, 976215. doi: 10.3389/fvets.2022.976215 36619963 PMC9813401

[7] Leong K.N., Chow T.S., Wong P.S., Hamzah S.H., Ahmad N., & Ch’ng C.C. (2015). Outbreak of human brucellosis from consumption of raw goats’ milk in Penang, Malaysia. *American Journal of Tropical Medicine and Hygiene*, 93(3), 539–41. doi: 10.4269/ajtmh.15-0246 26055742 PMC4559693

[8] WHO. 2011. Brucellosis in humans and animals. World Health Organization. Fact Sheet. https://www.who.int/news-room/fact-sheets/detail/brucellosis

[9] Ragan V.E. (2002). Animal and plant health inspection service. The animal and plant health inspection service (APHIS) brucellosis eradication program in the United States. *Veterinary Microbiology*, 90(1–4), 11–8. 10.1016/s0378-1135(02)00240-7 12414129

[10] Sriranganathan N., Seleem M.N., Olsen S.C., Samartino L.E., Whatmore A.M., Bricker B., et al. (2009). *Brucella*. In: Genome mapping and genomics in animal-associated microbes. Springer, Berlin. p. 1–64.

[11] Lai S., Zhou H., Xiong W., Gilbert M., Huang Z., Yu J., et al. (2017). Changing epidemiology of human brucellosis, China, 1955–2014. *Emerging Infectious Diseases*, 23(2), 184–194. doi: 10.3201/eid2302.151710 28098531 PMC5324817

[12] Ran X., Chen X., Wang M., Cheng J., Ni H., Zhang X-X., et al. (2018). Brucellosis seroprevalence in ovine and caprine flocks in China during 2000–2018: a systematic review and meta-analysis. *BMC Veterinary Research*, 14(1), 393. doi: 10.1186/s12917-018-1715-6 30541567 PMC6292006

[13] Musallam I.I., Abo-Shehada M.N., & Guitian J. (2015). Knowledge, attitudes, and practices associated with brucellosis in livestock owners in Jordan. *American Journal of Tropical Medicine and Hygiene*. 93(6), 1148–1155. doi: 10.4269/ajtmh.15-0294 26438029 PMC4674226

[14] NBS. (2018). China statistical yearbook. http://www.stats.gov.cn/sj/ndsj/2018/indexeh.htm

[15] Zeng J.Y., Ciren D.J., Yundan D.Z., Pu Q., Gongjue C.W., Jiumei D.J., et al. (2018). A study of the knowledge, attitudes and practices of Tibetan yak herders with respect to brucellosis. *International Health*. 10(4), 294–301. doi: 10.1093/inthealth/ihx076 29471484

[16] Mangalgi S.S., Sajjan A.G., Mohite S.T., Gajul S. (2016). Brucellosis in occupationally exposed groups. Journal of Clinical Diagnostic Research. 10(4), DC24–7. doi: 10.7860/JCDR/2016/15276.7673 27190804 PMC4866102

[17] Kiffner C., Latzer M., Vise R., Benson H., Hammon E., Kioko J. (2019). Comparative knowledge, attitudes, and practices regarding anthrax, brucellosis, and rabies in three districts of northern Tanzania. BMC Public Health. 19(1), 1625. doi: 10.1186/s12889-019-7900-0 31796011 PMC6889212

[18] Holt H.R., Eltholth M.M., Hegazy Y.M., El-Tras W.F., Tayel A.A., & Guitian J. (2011). *Brucella* spp. infection in large ruminants in an endemic area of Egypt: cross-sectional study investigating seroprevalence, risk factors and livestock owner’s knowledge, attitudes and practices (KAPs). *BMC Public Health*, 11, 341. 10.1186/1471-2458-11-341 21595871 PMC3121632

[19] Hussain S., Hussain A., Zia UuR. Naqvi S.M.R, Zahoor M.Y., Bilal M., HO J. et al. (2021). Knowledge, attitude, and practices associated with brucellosis among livestock owners and its public health impact in Punjab, Pakistan. *Biologia* 76, 2921–2929. 10.1007/s11756-021-00765-2

[20] Kozukeev T.B., Ajeilat S., Maes E., & Favorov M. (2006). Centers for Disease Control and Prevention (CDC). Risk factors for brucellosis—Leylek and Kadamjay districts, Batken Oblast, Kyrgyzstan, January-November 2003. *Morbidity and Mortality Weekly Report Supplement*, 55(1), 31–34. 16645580

[21] Sofian M., Aghakhani A., Velayati A.A., Banifazl M., Eslamifar A., & Ramezani A. (2008). Risk factors for human brucellosis in Iran: a case–control study. *International Journal of Infectious Diseases*, 12(2), 157–161. doi: 10.1016/j.ijid.2007.04.019 17698385

[22] Kothalawala K.A.C.H.A, Makita K., Kothalawala H., Jiffry A.M., Kubota S., & Kono H. (2018). Knowledge, attitudes, and practices (KAP) related to brucellosis and factors affecting knowledge sharing on animal diseases: a cross-sectional survey in the dry zone of Sri Lanka. *Tropical Animal Health and Production*, 50(5), 983–989. doi: 10.1007/s11250-018-1521-y 29392550

[23] Nabirye H.M., Erume J., Nasinyama G.W., Kungu J.M., Nakavuma J., Ongeng D., et al. (2017). Brucellosis: Community, medical and veterinary workers’ knowledge, attitudes, and practices in Northern Uganda. *International Journal of One Health*, 3, 12–18. https://www.onehealthjournal.org/Vol.3/3.html

[24] Peck M.E., Jenpanich C., Amonsin A., Bunpapong N., Chanachai K., Somrongthong R., et al. (2019). Knowledge, attitudes and practices associated with brucellosis among small-scale goat farmers in Thailand. *Journal of Agromedicine*. 24(1), 56–63. doi: 10.1080/1059924X.2018.1538916 30350754

[25] Lindahl E., Sattorov N., Boqvist S., & Magnusson U. (2015). A study of knowledge, attitudes and practices relating to brucellosis among small-scale dairy farmers in an urban and peri-urban area of Tajikistan. *PLoS One*, 10(2), e0117318. doi: 10.1371/journal.pone.0117318 25668783 PMC4323107

[26] Ritter C., Jansen J., Roche S., Kelton D.F., Adams C.L., & Orsel K. (2017). Invited review: Determinants of farmers’ adoption of management-based strategies for infectious disease prevention and control. *Journal of Dairy Science*, 100(5), 3329–3347. doi: 10.3168/jds.2016-11977 28237585

[27] TeshomeYimer B., Feleke B.E., Bogale K.A., & Tsegaye G.W. (2021). Factors associated with human brucellosis among patients attending in Ayu Primary hospital, North Showa, Ethiopia: a case control study. *Ethiopian Journal of Health Sciences*, 31(4), 709–718. doi: 10.4314/ejhs.v31i4.4 34703169 PMC8512956

[28] Arif S., Thomson P.C., Hernandez-Jover M., McGill D.M., Warriach H.M., & Heller J. (2017). Knowledge, attitudes and practices (KAP) relating to brucellosis in smallholder dairy farmers in two provinces in Pakistan. *PLoS One*, 12(3), e0173365. doi: 10.1371/journal.pone.0173365 28301498 PMC5354373

[29] Liu F., Gong Q-L., Zhang R., Chen Z-Y., Wang Q., Sun Y-H., et al. (2021). Prevalence and risk factors of bluetongue virus infection in sheep and goats in China: A systematic review and meta-analysis. *Microbial Pathogenesis*, 161, 105170. doi: 10.1016/j.micpath.2021.105170 34492305

[30] Manuja B.K., Manuja A., & Singh R.K. (2014). Globalization and livestock biosecurity. *Agricultural Research*. 3(1), 22–31. doi: 10.1007/s40003-014-0097-7 34262883 PMC7149119

[31] Cloete A., Gerstenberg C., Mayet N., & Tempia S. (2019). Brucellosis knowledge, attitudes and practices of a South African communal cattle keeper group. *Onderstepoort Journal of Veterinary Research*, 86(1), e1–e10. doi: 10.4102/ojvr.v86i1.1671 30843408 PMC6407466

[32] Zhang N., Zhou H., Huang D.S., & Guan P. (2019). Brucellosis awareness and knowledge in communities worldwide: A systematic review and meta-analysis of 79 observational studies. *PLOS Neglected Tropical Diseases*, 13(5), e0007366. doi: 10.1371/journal.pntd.0007366 31048848 PMC6497230

[33] Mligo B.J., Sindato C., Yapi R.B., Mathew C., Mkupasi E.M., Kazwala R.R., et al. (2022). Knowledge, attitude and practices of frontline health workers in relation to detection of brucellosis in rural settings of Tanzania: a cross-sectional study. *One Health Outlook*. 4(1), 1. doi: 10.1186/s42522-021-00056-5 34983693 PMC8725462

[34] Mohamed M.M.G., Shwaib H.M., Fahim M.M., Ahmed E.A., Omer M.K., Monier I.A., et al. (2017). Ebola hemorrhagic fever under scope, view of knowledge, attitude and practice from rural Sudan in 2015. *Journal of Infection and Public Health*, 10(3), 287–294. doi: 10.1016/j.jiph.2016.05.016 27396613

[35] Tulu D. (2022) Bovine brucellosis: epidemiology, public health implications, and status of brucellosis in Ethiopia. *Veterinary medicine (Auckland*, *N*.*Z*.*)*, 13, 21–30. doi: 10.2147/VMRR.S347337 35028300 PMC8752066

[36] Pappas G., Siozopoulou V., Saplaoura K., Vasiliou A., Christou L., Akritidis N., et al. (2007). Health literacy in the field of infectious diseases: the paradigm of brucellosis. *Journal of Infection*, 54(1), 40–45. doi: 10.1016/j.jinf.2006.01.018 16533534

[37] Al-Shamahy H., Whitty C., & Wright S. (2000). Risk factors for human brucellosis in Yemen: a case control study. *Epidemiology and Infection*, 125(2), 309–313. doi: 10.1017/s0950268899004458 11117954 PMC2869603

[38] Alsaleh F.M., Elzain M., Alsairafi Z.K., & Naser A.Y. (2023). Perceived knowledge, attitude, and practices (KAP) and fear toward COVID-19 among patients with diabetes attending primary healthcare centers in Kuwait. *International Journal of Environmental Research and Public Health*, 20(3), 2369. doi: 10.3390/ijerph20032369 36767736 PMC9916070

[39] Azim M.R., Ifteakhar K.M.N., Rahman M.M., & Sakib Q.N. (2023) Public knowledge, attitudes, and practices (KAP) regarding antibiotics use and antimicrobial resistance (AMR) in Bangladesh. *Heliyon*, 9(10), e21166. doi: 10.1016/j.heliyon.2023.e21166 37916103 PMC10616402

